# Investigating the Role of Anti-TPO Antibodies in HIV-Associated Thrombocytopenia before and after Initiation of HAART: A Case-Control Longitudinal Study

**DOI:** 10.3390/v15112226

**Published:** 2023-11-08

**Authors:** Aristotelis Tsiakalos, John G. Routsias, Georgios Schinas, Sarah Georgiadou, Nikolaos V. Sipsas, Karolina Akinosoglou

**Affiliations:** 1 Leto General, Maternity & Gynecology Clinic, 11524 Athens, Greece; atsiakalos@gmail.com; 2 Department of Microbiology, Medical School, National and Kapodistrian University of Athens, 11527 Athens, Greece; jroutsias@yahoo.com; 3 School of Medicine, University of Patras, 26504 Rio, Greece; georg.schinas@gmail.com; 4 Department of Medicine and Research Laboratory of Internal Medicine, General University Hospital of Larissa, 41110 Larissa, Greece; s_georgiadou@hotmail.com; 5 Laiko General Hospital of Athens and Medical School, National and Kapodistrian University of Athens, 11527 Athens, Greece; nsipsas@med.uoa.gr; 6 Department of Internal Medicine and Infectious Diseases, University General Hospital of Patras, 26504 Rio, Greece

**Keywords:** HIV-1, thrombocytopenia, anti-thrombopoietin antibodies, highly active antiretroviral therapy (HAART), platelet count

## Abstract

This longitudinal, case-control study aimed to investigate the role of thrombopoietin (TPO) and anti-TPO antibodies in HIV-associated thrombocytopenia, focusing on the changes seen before and after the initiation of highly active antiretroviral therapy (HAART). Patients were assessed before and at least six months after the initiation of HAART. In total, 75 PLWHIV (age/sex-matched and randomized at 2:1, according to thrombocytopenia status) were included in this study. The baseline assessment revealed significantly higher TPO levels in thrombocytopenic patients (140.45 vs. 106.8 mg/mL, *p* = 0.008). Furthermore, anti-TPO-positive patients displayed lower platelet counts (109,000 vs. 139,000/L, *p* = 0.002) and TPO levels (114.7 vs. 142.7 mg/mL, *p* = 0.047). Longitudinally, HAART initiation reduced the frequency of thrombocytopenia from 75.47% to 33.96% (*p* < 0.001) and elevated the median platelet counts from 131,000 to 199,000 (*p* < 0.001). No significant difference in median platelet counts was found post-HAART among the anti-TPO subgroups (*p* = 0.338), a result contrasting with pre-HAART findings (*p* = 0.043). Changes in anti-TPO status corresponded with significant platelet count alterations (*p* = 0.036). Notably, patients who became anti-TPO negative showed a median increase of 95,000 platelets (IQR: 43,750–199,500). These marked differences between subgroups underscore the potential role of anti-TPO antibodies in modulating the hematological response to HAART. Further research is needed to elucidate the complex interplay between HIV infection, HAART, and thrombocytopenia.

## 1. Introduction

Acquired immunodeficiency syndrome (AIDS) is characterized by the severe impairment and progressive damage of both cellular and humoral immune responses. In addition to the immunological complications, infection with the human immunodeficiency virus (HIV) leads to altered hematopoiesis, affecting all three cell lines: red blood cells, white blood cells, and platelets [1]. Thrombocytopenia, a condition defined as a platelet count of less than 150,000/mm^3^ in a blood panel, is, in fact, the second most frequent hematologic disorder in patients infected with HIV, following anemia [2], occurring in about 4% to 26% of patients living with HIV (PLWHIV) [3]. It can occur independently of other cytopenias and at all stages of HIV infection [4], sometimes presenting as its first manifestation [5]. The degree of thrombocytopenia is generally mild to moderate; however, severe reduction involving a platelet count of < 50,000/mL also occurs [6]. Moreover, the platelet count has been found to correlate with the circulating CD4 count and the viral load [7].

Highly active antiretroviral therapy (HAART) has been shown to improve the hematological parameters, including platelet counts, in PLWHIV [8]. HAART’s role in restoring platelet count may be attributed to decreasing the incidence of opportunistic infections and enhancing hematopoietic progenitor cell growth by reducing the viral load through the inhibition of viral replication [9]. Although HAART is extensively utilized, thrombocytopenia remains a significant clinical concern. A recent systematic review has quantified the pooled prevalence of thrombocytopenia following HAART initiation at 11.64%, with a 95% confidence interval ranging from 6.66% to 16.62% [3]. Therefore, it is evident that further research is needed to better understand the complex interplay between HIV infection, HAART, and thrombocytopenia.

**Figure 1:**
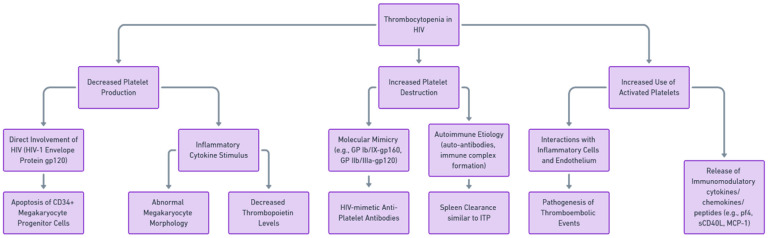
Pathophysiological pathways contributing to thrombocytopenia in HIV. Note: The flowchart summarizes the key pathophysiological pathways implicated in thrombocytopenia among HIV-infected individuals, highlighting the complex interplay between viral factors and host immune responses. (Abbreviations: HIV, human immunodeficiency virus; ITP, immune thrombocytopenia; CD34, cluster of differentiation 34; gp/GP, glycoprotein; sCD40L, soluble CD40 ligand; MCP-1, monocyte chemoattractant protein-1; pf4, platelet factor 4).

The pathogenesis of HIV-associated thrombocytopenia is multifactorial, involving both direct and indirect mechanisms and potentially leading to thrombotic complications. Direct mechanisms include the infection of megakaryocytes, the progenitor cells of platelets, by the virus, leading to impaired platelet production [10]. Indirect mechanisms involve immune-mediated ways of platelet destruction, including the production of anti-platelet antibodies, immune complex deposition on platelets, and the effects of opportunistic infections and certain drugs [11]. The potential impairment of platelet generation has been suggested as one of the underlying factors in HIV-related thrombocytopenia, a theory supported by observations of platelet dynamics, outcomes from tests examining megakaryocyte colony formation, and electron microscopy revealing the direct viral infection of megakaryocytes [12]. Figure 1 summarizes the pathways of HIV-associated thrombocytopenia. 

Thrombopoietin has been demonstrated to serve as the chief regulator of platelet generation. TPO is a glycoprotein hormone that stimulates the production and differentiation of megakaryocytes. According to the existing framework governing the levels of circulating TPO, it is postulated that TPO is continually produced by the liver, kidney, and bone marrow, and its removal from the bloodstream is facilitated by mpl receptors (with the mpl ligand identified as endogenous plasma TPO) located on platelets and megakaryocytes through mass action [13,14,15]. In human research, compelling evidence exists indicating that serum TPO levels exhibit an inverse correlation with the mass of both platelets and megakaryocytes. In the context of thrombocytopenia, synthetic analogs of TPO, such as romiplostim and eltrombopag, are being used clinically to stimulate platelet production.

That said, one plausible contributory factor that has yet to be explored in the context of HIV-associated thrombocytopenia is the role of anti-thrombopoietin (anti-TPO) antibodies, which could mediate platelet destruction or impair platelet production. The presence of anti-TPO antibodies has been observed in patients with immune thrombocytopenia (ITP). In a study of 101 patients with ITP, 23.8% of patients were found to have anti-thrombopoietin antibody reactivity [16]. The presence of anti-TPO antibodies has also been reported in patients treated with the recombinant forms of TPO (rhTPO) as well as erythropoietin (rhEPO) [17,18]. To the best of our knowledge, no previous study has examined the relationship between anti-TPO antibodies and thrombocytopenia among PLWHIV, particularly in the context of HAART initiation. To address this gap, the present study aims to investigate the longitudinal changes in anti-TPO antibody levels in a cohort of HIV-1 patients before and six months after the initiation of HAART. Utilizing a case-control design, this study will also explore whether the presence of anti-TPO antibodies correlates with the incidence or severity of thrombocytopenia.

## 2. Materials and Methods

### 2.1. Study Design and Population

This article reports a case-control longitudinal study conducted within the Infectious Disease Unit of the Laiko General Hospital of Athens between 2019 and 2020. Upon written informed consent, PLWHIV were recruited during their first visits, at which point they were treatment-naïve. A subsequent visit was made 6 months after the initiation of HAART. The study conformed to the ethical guidelines of the 1975 Declaration of Helsinki and received approval from the Institutional Review Board and the respective Ethics Committee of the Laiko General Hospital of Athens, Greece.

### 2.2. Inclusion-Exclusion Criteria

Participants were included in this study upon written informed consent and confirmation of HIV (+) status. Prior to inclusion, patients were tested for acute or chronic infectious diseases affecting platelet count, including EBV and CMV, and Helicobacter pylori, Plasmodium, Leishmania, and Babesia infections. In the case of a positive test, patients were excluded from this study. Moreover, patients with evidence of neoplasm complicated by disseminated intravascular coagulation or bone suppression/infiltration (e.g., lymphoma, leukemia, or solid tumors), chronic inflammatory/autoimmune diseases (e.g., systemic lupus erythematosus or rheumatoid arthritis), pregnancy, evidence of chronic disease with associated impaired liver function or hypersplenism, alcohol overconsumption, nutrient deficiency (e.g., vitamin B12, folate, or copper), a history of hereditary thrombocytopenia (e.g., Bernard–Soulier syndrome, Wiskott–Aldrich syndrome, Alport syndrome, von Willebrand disease, etc.), aplastic anemia, paroxysmal nocturnal hemoglobinuria, thrombotic microangiopathy, antiphospholipid syndrome, or evidence of drug-induced thrombocytopenia (e.g., from heparin, sulfonamide, vancomycin, ibuprofen, or glycoprotein IIb/IIIa inhibitors) were also excluded from this analysis.

### 2.3. Definitions

Thrombocytopenia was defined as a platelet count of < 150,000/mm^3^. The age and sex of the participants were matched to control for these variables. AIDS was defined as a CD4 count of < 250 cells/mm^3^, in conjunction with an AIDS-defining condition.

### 2.4. Sampling and Laboratory Methods

Whole-blood samples were obtained during the first visit and at least 6 months following HAART initiation. The samples were differentially centrifuged and stored at −70 °C until testing. The serum levels of TPO were quantified, employing a commercially available Quantikine Human TPO Immunoassay (R&D Systems, Minneapolis, MN, USA). This assay is a solid-phase enzyme-linked immunosorbent assay (ELISA) based on the sandwich principle.

To assess the presence of circulating anti-TPO IgG antibodies, a modified commercial ELISA assay, designed originally for the measurement of TPO (Quantikine; R&D Systems), was utilized. In preparation for the assay, anti-TPO precoated ELISA plates were saturated with 80 ng/mL of recombinant human TPO antigen (recTPO; R&D Systems), in 0.1% bovine serum albumin (BSA)/phosphate-buffered saline (PBS), pH = 7.2 (200 µL/well) during 1 h of incubation at 37 °C. The coating protocol, including the concentration of recombinant TPO, was determined through a series of optimization experiments. The ELISA plates saturated with recombinant TPO were then washed three times with the kit’s wash buffer and incubated for 90 min with 200 µL/well of diluted sera (1:100 in 0.1% BSA/PBS, pH = 7.2). After washing away any unbound substances (7 times), anti-human IgG, conjugated with alkaline phosphatase (Jackson Immunoresearch, West Grove, PA, USA), was added at a dilution of 1:1000 in 0.1% BSA/PBS, at pH = 7.2. Following 9 washes with the kit’s wash buffer, para-nitro-phenyl-phosphate (PNPP) substrate solution (Sigma-Aldrich, St Louis, MO, USA) was added and the color was measured at 405 nmA, a positive cutoff value for the assay was established, based on the mean optical density (OD) plus 3 standard deviations derived from a cohort of 30 healthy controls.

### 2.5. Data Analysis

Descriptive statistics were used to summarize the baseline characteristics. Continuous variables are reported as median values, accompanied by their interquartile ranges (IQRs) as indices of dispersion; these ranges are specified as the 25th to 75th percentiles. Categorical variables are described using frequencies and percentages. The Shapiro–Wilk test was used to assess the normality of the continuous variables. For a comparison of the continuous variables, Student’s *t*-test or the Mann–Whitney U test were used. Categorical variables were compared using the chi-square test or Fisher’s exact test. The associations between variables were statistically assessed utilizing Spearman’s rank correlation coefficient. Longitudinal changes in the continuous variables were assessed using either the Wilcoxon signed-rank test or the paired-sample *t*-test. For evaluating longitudinal alterations in the paired proportions among subgroups, the McNemar change test was utilized. The Kruskal–Wallis test was employed to assess the overall differences in the changes in platelet counts across various anti-TPO change categories: the persistence of negativity, change to positivity, change to negativity, and persistence of positivity. Following the Kruskal–Wallis test, pairwise comparisons were conducted using Dunn’s post hoc test, along with the Holm–Bonferroni correction for multiple comparisons, to identify which subgroups contributed to the observed differences. A significance level of *p* < 0.05 was employed for all tests. Statistical analyses were conducted using Python, utilizing scientific libraries such as SciPy and Pandas, and SPSS Version 28 (IBM Corp., Armonk, NY, USA).

## 3. Results

### 3.1. Overview of the Study Population

The study population consisted of 75 HIV-1 patients with a median age of 37.0 years (IQR: 32.0–46.0), with the majority being Caucasian (92.0%) and male (82.7%). The median platelet count was 131,000 × 10^9^/L (IQR: 99,000–165,000), and the median CD4 count was 221 cells/µL (IQR: 69–437). The median viral load was 173,537 copies/mL (IQR: 32,992–430,904), with 53.3% of the total study population having been diagnosed with AIDS. The median TPO level was 129.2 mg/mL (IQR: 101.9–223.4) and 33.3% of the patients were anti-TPO positive, as previously intended (Table 1).

A weak negative, yet non-significant, correlation between thrombopoietin (TPO) levels and platelet counts (rho = −0.199, *p* = 0.088) was noted.

### 3.2. HIV Disease Severity Is Associated with Thrombocytopenia

In the comparison between patients with and without thrombocytopenia, defined as a platelet count of <150,000 mm^3^,there were no significant differences in age, sex, ethnicity, or most clinical characteristics (Table 2). A significantly higher proportion of patients with thrombocytopenia had AIDS (64.8%) compared to those without thrombocytopenia (23.8%) (*p* = 0.002). In terms of HIV staging, there were significant differences in the distribution across stages B1–B3 (29.7% vs. 14.3%, *p* = 0.002) and C1–C3 (37% vs. 9.5%, *p* < 0.001), with a higher proportion of patients with thrombocytopenia in these advanced stages. Furthermore, patients with thrombocytopenia had significantly lower CD4 counts (median: 163 cells/µL, IQR: 64.25–313.75) compared to those without thrombocytopenia (median: 441 cells/µL, IQR: 251–672) (*p* = 0.004). However, patients with thrombocytopenia had significantly higher TPO levels (median: 140.45 mg/mL, IQR: 112.15–248.08) compared to those without thrombocytopenia (median: 106.8 mg/mL, IQR: 93.3–142.7) (*p* = 0.008).

No correlation was detected between TPO levels and platelet counts among the thrombocytopenia subgroups.

### 3.3. Anti-TPO Positive (+) PLWHIV Present Lower Platelet Counts and Lower TPO Levels

When comparing anti-TPO positive (anti-TPO (+)) and anti-TPO negative (anti-TPO (−)) patients, there were no significant differences in age, sex, ethnicity, or co-morbidities such as HCV, HBV, liver disease, neoplasia, or splenomegaly (Table 3). The difference in the proportion of patients with AIDS was not statistically significant (60.0% vs. 50.0%, *p* = 0.567). Anti-TPO (+) patients had significantly lower platelet counts (median: 109,000 × 10^9^/L, IQR: 93,000–124,000) compared to anti-TPO (−) patients (median: 139,000 × 10^9^/L, IQR: 128,250–171,500) (*p* = 0.002). Similarly, anti-TPO (+) patients had significantly lower TPO levels (median: 114.7 mg/mL, IQR: 97.8–136.1) compared to anti-TPO (−) patients (median: 142.7 mg/mL, IQR: 106.8–240.3) (*p* = 0.047).

In the anti-TPO (−) subgroup, a significant negative correlation between TPO levels and platelet counts was detected (rho = −0.628; *p*-value of < 0.001) Conversely, in the anti-TPO (+) subgroup, a significant positive correlation was observed (rho = 0.432; *p*-value = 0.031).

### 3.4. HAART Initiation Reduces the Frequency of Thrombocytopenia and Anti-TPO Positivity

The longitudinal part of the study evaluated the impact of HAART on various hematological and immunological parameters at two time points—before and after the initiation of HAART (Table 4). Only 53 patients underwent the second sampling following HAART initiation. A significant reduction in the frequency of thrombocytopenia was observed, declining from 75.47% to 33.96% (*p* < 0.001). The frequency of anti-TPO positivity also decreased from 28.30% to 16.98%, although this change was not statistically significant (*p* = 0.058). A focused examination of a thrombocytopenia-affected subgroup, consisting of 40 individuals, yielded similar results, further confirming the absence of temporal fluctuations in anti-TPO prevalence related to HAART initiation (*p* = 0.109).

### 3.5. Platelet Counts Significantly Increase in Anti-TPO (−) Patients but Not Markedly in Anti-TPO (+) Patients after HAART Introduction, Reaching Comparable Levels in Both Groups

**Figure 2:**
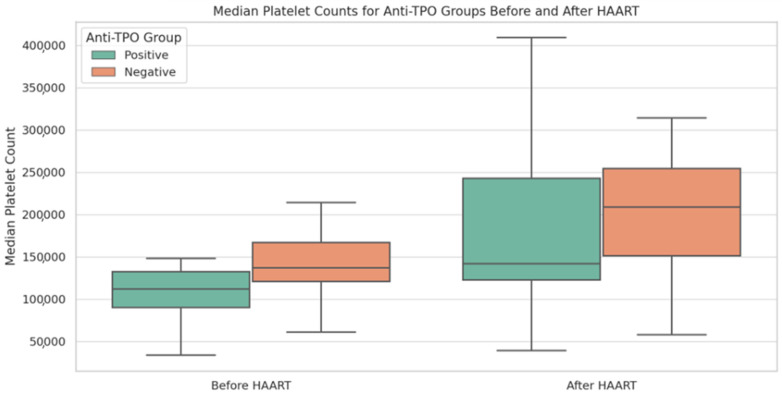
Median platelet counts for anti-TPO groups before and after HAART initiation. The box plots above visualize the distribution (median and IQR) of platelet counts for each anti-TPO group across two time points.

In this subgroup investigation, we assessed the role played by the presence of anti-TPO antibodies at baseline on platelet counts at the two time points. Within the anti-TPO (+) group, a marginally significant alteration in median platelet counts was noted between the two time points—before and after HAART initiation (*p* = 0.048)—with a median increase of 51,000 (IQR: (−5000) to 112,000). In contrast, the anti-TPO (−) group displayed a highly significant difference in platelet counts across the time points (*p* < 0.001), with a similar median increase in the platelet count (48,500 [IQR: (−2000) to 101,500]). A significant difference in median platelet counts between the anti-TPO (+) and anti-TPO (−) groups was noted at time point 1 (*p* = 0.043), but not at time point 2 following HAART initiation (*p* = 0.338). These results are visually summarized in Figure 2.

### 3.6. Anti-TPO Seroconversion following HAART Initiation Was Associated with a Subsequent Significant Change in Platelet Levels

Building upon these findings, we then broadened our inquiry to assess the overall differences in platelet counts across various anti-TPO change categories: persistence of negativity, change to positivity, change to negativity, and persistence of positivity. A significant overall difference in platelet count changes across the four anti-TPO change categories was revealed (*p* = 0.036). The median changes in platelet counts across each anti-TPO change category are shown in Table 5.

**Figure 3:**
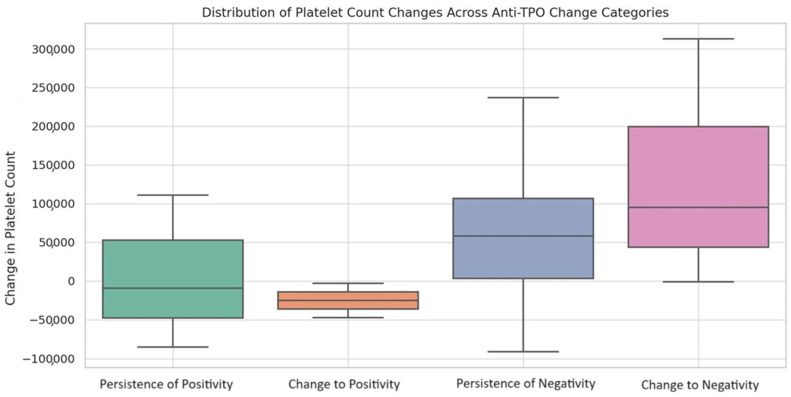
Distribution of platelet-count changes across anti-TPO change categories. The box plots above visualize the distribution of platelet count changes across each anti-TPO change category. The plots include whiskers that extend to 1.5 times the interquartile range, providing a more detailed view of the data distribution.

Notably, patients who changed to anti-TPO negativity showed a median increase of 95,000 platelets (×10^9^/L) [IQR: 43,750–199,500], while those who changed to anti-TPO positivity showed a median decrease of 25,000 platelets (×10^9^/L) [IQR: −36,000–(−14,000)]. Post hoc testing indicated statistically significant differences between all pairs of categories (*p* < 0.001) (Figure 3).

### 3.7. Anti-TPO Prevalence Was Not Associated with Severity of Thrombocytopenia

To elucidate the longitudinal changes in anti-TPO antibody status in relation to thrombocytopenia severity, a test for paired proportions was conducted. The analysis was stratified by the four pre-defined thrombocytopenia severity categories: < 50 k, 50–99 k, 100–150 k, and ≥ 150 k platelet counts.

**Figure 4:**
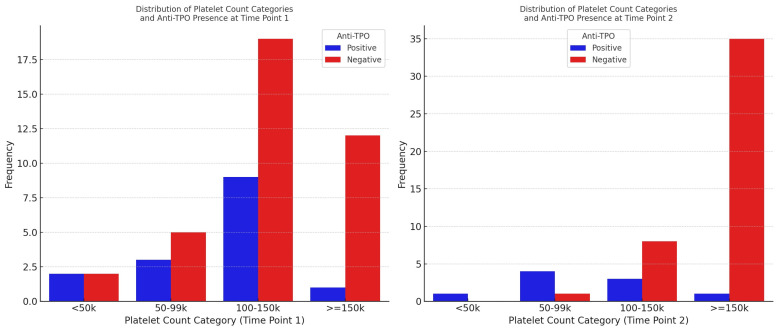
Proportions of anti-TPO subgroups across the thrombocytopenia severity categories at both time points.

The distribution of thrombocytopenia severity in relation to anti-TPO antibody status at two time points is shown in Figure 4.

Across all the categories of thrombocytopenia severity, no statistically significant changes in the proportions of anti-TPO (+) cases were observed between the two time points (Table 6). When considering thrombocytopenia as a binary variable (platelet count < 150,000), the test for paired proportions revealed no statistically significant change in the proportion of anti-TPO (+) and anti-TPO (−) from time point 1 to time point 2 (*p* = 0.392).

## 4. Discussion

To the knowledge of the current authors, this is the first study assessing the role of anti-TPO in HIV-associated thrombocytopenia and its fluctuation following HAART initiation. We showed that thrombocytopenia is associated with HIV disease severity, which, however, is restored following HAART introduction. Anti-TPO (+) PLWHIV present significantly increased thrombocytopenia and lower TPO levels compared to the anti-TPO (−) group of patients. HAART initiation results in an increase in platelet counts, an effect that is stronger in anti-TPO (−) PLWHIV. Moreover, antiretroviral therapy shows a marginal trend of decreasing anti-TPO (+) prevalence; however, seroconversion does not seem to be related to the severity of the initial thrombocytopenia.

We have shown, among other findings, that HIV disease severity is reflected in CD counts and viral load and is associated with thrombocytopenia, while the levels are restored following HAART introduction. A noteworthy and positive connection between the blood platelet count and the CD4 count has been widely noted in the past [19,20]. Taremwa and the research team in Uganda reported a significant link between the blood platelet count and CD4 T cell lymphocyte count (*p* < 0.05) [20]. Conversely, a substantial and adverse correlation emerged between the blood platelet count and plasma viral load [20]. This outcome aligns perfectly with the findings of O’Bryan et al. [21] in the United States in 2015, who likewise observed an inverse relationship between platelet count and HIV viral load. Several other studies have indicated that HAART introduction leads to a reduction in the prevalence of thrombocytopenia as a result of immune reconstitution, as well as viral suppression [3,8,22]. However, there are also significant reports, as Vannappagari et al. highlighted in 2011, documenting the continued occurrence of this hematological abnormality in patients receiving HAART [23]. The impact of HAART on platelets can vary, either positively or negatively, generally depending on the specific combination of drugs used, since many drugs employed in the treatment of HIV-related conditions possess myelosuppressive properties. Nonetheless, severe cytopenia is most commonly associated with the use of zidovudine, as noted by Behler et al. in 2005 [24].

Approximately one-third of PLWHIV present with anti-TPO (+) in our study. Previous investigations identified the presence of non-organ-specific antibodies in 20% to 45% of PLWHIV [25]. The potential mechanisms contributing to the production of these antibodies in the context of HIV infection encompass molecular mimicry [26,27,28], disruptions in the interplay between B and T lymphocytes [29], and the activation of polyclonal B lymphocytes. One hypothesis suggests that anti-TPO may be an autoantibody triggered by chronic inflammation [30]. It is postulated that misfolded proteins forming complexes with MHC class II molecules could be identified as “neo-self” antigens by immune cells, subsequently stimulating the production of autoantibodies [31]. However, whether those antibodies remain functional or what their potential clinical impact may be remains elusive.

Nonetheless, we have shown that anti-TPO (+) PLWHIV present lower platelet counts and TPO levels. Thrombocytopenia resulting from reduced platelet production is linked to heightened serum TPO levels, whereas in cases of platelet destruction, TPO levels do not show any elevation [12]. Because TPO is constitutively produced, the appearance of a neutralizing antibody to TPO seems to reduce platelet production by 95%; the effect is similar to that seen in animals in which the TPO gene has been knocked out or in which autoantibodies to TPO have been generated [32,33,34]. Hence, our study possibly suggests a new mechanism for thrombocytopenia in PLWHIV, one that is mediated by but not limited to anti-TPO production. Similar to HIV, the presence of anti-TPO and its implications have been explored in systemic erythematosus lupus (SLE) [35,36]. Autoantibodies against TPO have been detected in 23–39% of SLE patients [35,37]; however, as is similar to our case, their exact role in the pathogenesis of thrombocytopenia remains unknown, even though they seem to contribute to increased peripheral platelet turnover and a reduction in the effective circulating TPO. Similarly, in the SLE study, anti-TPO antibodies were associated with lower circulating TPO levels and lower mean platelet values at long-term follow-up. HAART significantly improved the platelet counts and reduced the prevalence of thrombocytopenia. In the SLE study, it was found that post-thrombocytopenic individuals who had received more intense immunosuppression showed restored normal platelet counts and did not exhibit autoantibody activity against TPO. In a similar study, Fukuda et al. examined the presence and role of anti-TPO in patients with diabetes mellitus (DM) [30]. While this study did not allow the authors to establish a causal link between the presence of anti-TPO antibodies and a reduced platelet count, a prior report did suggest the direct impact of anti-THPO antibodies on thrombocytopenia [17]. In that particular study, it was proposed that the anti-TPO antibodies could have hindered the regular functioning of thrombopoietin, consequently leading to a decrease in platelet production in DM patients.

We observed that HAART initiation reduces the frequency of thrombocytopenia and anti-TPO positivity. One possible explanation would be that the lower anti-TPO antibody seropositivity is a result of the HAART-induced reduction in HIV-associated immune dysregulation, leading to a decrease in the production of autoantibodies, including anti-TPO antibodies. The perspective that immune dysregulation improves with the suppression of viral replication through HAART, albeit not entirely, gains additional support from the discovery of a link between the presence of non-organ-specific antibodies and less effective immunological control [38]. These findings are consistent with another study that demonstrated a reduction in autoantibody responses following the introduction of HAART [39]. The noted phenomenon wherein the restoration of cellular immune function leads to the reduced production of autoantibodies offers valuable insights into the potential involvement of T cells in the development of autoimmunity [38].

The platelet counts showed a marked increase in anti-TPO (−) but not anti-TPO (+) patients after HAART introduction, reaching comparable levels in both groups. It seems that HAART, by suppressing HIV replication and reducing the virus’s direct myelotoxic effects, facilitates an increase in platelet counts in the presence of increased TPO levels, particularly in anti-TPO (−) PLWHIV. On the contrary, even though platelet counts increase in anti-TPO (+) PLWHIV, it appears that low TPO levels do not allow for marked platelet elevation, maintaining mild pressure on platelet production, indicating the presence of multiple pathways associated with TPO-related thrombocytopenia. We also observed that anti-TPO seroconversion following HAART was associated with a significant change in platelet levels. This implies that the presence of anti-TPO antibodies may have a direct impact on platelet counts. However, it is interesting to note that anti-TPO seropositivity was not associated with the severity of thrombocytopenia in our study. This could suggest that while anti-TPO antibodies may affect platelet counts, other factors, such as the overall immune status of the patient and the degree of HIV-associated immune dysregulation, may play a more significant role in determining the severity of thrombocytopenia.

Our study has a number of limitations. First, the study is constrained by its relatively small sample size of 75 patients, which may limit the generalizability of the findings. Second, the study is single-center in design, thereby possibly not capturing regional and healthcare system-based variations. Moreover, the study faced the common challenges associated with longitudinal research, including participant attrition, which may have introduced a selection bias to the findings. Additionally, the gender distribution of patients was skewed toward males, potentially limiting the applicability of the results to female patients. Our study also lacked a control group of either healthy, HIV-negative individuals, or PLWHIV who were already on an established HAART regimen before participating in the study. This makes it difficult to determine the specific impact of HAART on the parameters measured in our study, including anti-TPO positivity. Future studies should consider including appropriate control groups to confirm and extend our findings. Our patients did not undergo a bone marrow biopsy, which means that we could not analyze the potential relationship between bone marrow megakaryocytes and the anti-TPO antibody or other relevant clinical parameters. Furthermore, no correlation was assessed regarding the type of HAART that was introduced. Even though the current regimens have not been strongly associated with thrombocytopenia, a potential effect similar to AZT remains unknown [40]. In addition, the ELISA assays used to detect the anti-TPO antibody provided only a qualitative assessment. Consequently, we were unable to establish a clear connection between platelet count and anti-TPO antibody titers. While the Quantikine Human TPO Immunoassay is well-validated, the modified ELISA for anti-TPO antibodies could introduce an element of variability that may impact the reproducibility of the results. Lastly, we did not assess the platelet-associated IgG levels due to the technical challenges of measuring them using frozen serum samples. Consequently, we cannot completely rule out the possibility of immune thrombocytopenia (ITP) in our study population.

While our study provides valuable insights into the role of anti-TPO antibodies in HIV-associated thrombocytopenia, it also highlights the need for further research in this area. In particular, it would be interesting to measure TPO levels in these patients following HAART initiation to better understand the interplay between TPO, anti-TPO antibodies, and platelet counts. Unfortunately, this was not possible in our study. Future studies should consider including this measurement, in addition to incorporating well-designed control groups, to construct a more comprehensive and robust comparative picture. A better understanding of these fundamental mechanisms could pave the way for novel therapeutic interventions and innovative approaches in tackling HIV-associated thrombocytopenia.

## Figures and Tables

**Table 1:** Population characteristics (*n* = 75).

**Epidemiology**	
Age (years), median (IQR)	37.0 (32.0–46.0)
Female sex, *n* (%)	13 (17.3%)
Caucasian (White), *n* (%)	69 (92.0%)
**Comorbidities**	
HCV (+), *n* (%)	6 (8.0%)
HBV (+), *n* (%)	5 (6.7%)
HBV/HCV coinfection, *n* (%)	3 (4.0%)
Liver disease, *n* (%)	8 (10.7%)
Neoplasia, *n* (%)	3 (4.0%)
Splenomegaly, *n* (%)	3 (4.0%)
**HIV Characteristics**	
AIDS, *n* (%)	40 (53.3%)
HIV stage, *n* (%)	
A1–A3	34 (45.3%)
B1–B3	19 (25.3%)
C1–C3	22 (29.3%)
**Laboratory Values**	
TPO (mg/mL), median (IQR)	129.2 (101.9–223.4)
Anti-TPO (+), *n* (%)	25 (33.3%)
Platelets (×10^9^/L), median (IQR)	131,000 (99,000–165,000)
Hemoglobin (g/dL), median (IQR)	13.0 (11.7–14.2)
White blood cells (×10^9^/L), median (IQR)	4900 (3400–5700)
CD4 count (cells/µL), median (IQR)	221 (69–437)
Viral load (copies/mL), median (IQR)	173,537 (32,992–430,904)

Abbreviations: *n*: Number of participants in each group; IQR: interquartile range; HCV: hepatitis C virus; HBV: hepatitis B virus; HIV: human immunodeficiency virus; AIDS: acquired immunodeficiency syndrome; ITP: immune thrombocytopenia; TPO: thrombopoietin; CD4: cluster of differentiation 4.

**Table 2:** Characteristics of participants with and without thrombocytopenia.

Variable	Thrombocytopenia (*n* = 54)	No Thrombocytopenia (*n* = 21)	*p*-Value
**Epidemiology**			
Age (years), median (IQR)	37.5 (31.0–45.5)	37.0 (33.0–47.0)	0.666
Female sex, *n* (%)	7 (13.0%)	6 (28.6%)	0.113
Caucasian (White), *n* (%)	49 (90.7%)	20 (95.2%)	1.000
**Comorbidities**			
HCV (+), *n* (%)	4 (7.4%)	2 (9.5%)	0.773
HBV (+), *n* (%)	5 (9.3%)	0 (0.0%)	0.156
HBV/HCV coinfection, *n* (%)	3 (5.6%)	0 (0.0%)	0.281
Liver disease, *n* (%)	7 (13.0%)	1 (4.8%)	0.310
Neoplasia, *n* (%)	3 (5.6%)	0 (0.0%)	0.281
Splenomegaly, *n* (%)	3 (5.6%)	0 (0.0%)	0.281
**HIV Characteristics**			
AIDS, *n* (%)	35 (64.8%)	5 (23.8%)	0.002
HIV stage, *n* (%)			
A1–A3	18 (33.3%)	16 (76.2%)	0.846
B1–B3	16 (29.7%)	3 (14.3%)	0.002
C1–C3	20 (37%)	2 (9.5%)	<0.001
**Laboratory Values**			
TPO (mg/mL), median (IQR)	140.45 (112.15–248.08)	106.8 (93.3–142.7)	0.008
Anti-TPO (+), *n* (%)	23 (42.59%)	2 (9.52%)	0.014
Platelets (×10^9^/L), median (IQR)	112,500 (90,000–134,500)	185,000 (170,000–264,000)	<0.001
Hemoglobin (g/dL), median (IQR)	12.85 (11.7–14.3)	13.5 (12.0–14.1)	0.628
White blood cells (×10^9^/L), median (IQR)	4400 (3055–5478)	5500 (4600–6530)	0.003
CD4 count (cells/µL), median (IQR)	163 (64.25–313.75)	441 (251–672)	0.004
Viral load (copies/mL), median (IQR)	153,995 (35,584–430,904)	185,137 (8215–421,823)	0.611

Abbreviations: *n*: Number of participants in each group; IQR: interquartile range; HCV: hepatitis C virus; HBV: hepatitis B virus; HIV: human immunodeficiency virus; AIDS: acquired immunodeficiency syndrome; ITP; immune thrombocytopenia; TPO: thrombopoietin, CD4: cluster of differentiation 4.

**Table 3:** Comparison of anti-TPO positive and negative groups.

	Anti-TPO Positive (*n* = 25)	Anti-TPO Negative (*n* = 50)	*p*-Value
Epidemiology			
Age (years), median (IQR)	39.0 (33.0–47.0)	37.0 (31.25–44.0)	0.412
Female sex, *n* (%)	4 (16.0%)	9 (18.0%)	1.000
Caucasian (White), *n* (%)	46 (92.0%)	23 (92.0%)	1.000
Comorbidities			
HCV (+), *n* (%)	3 (12.0%)	3 (6.0%)	0.652
HBV (+), *n* (%)	3 (12.0%)	2 (4.0%)	0.413
HBV/HCV coinfection, *n* (%)	2 (8.0%)	1 (2.0%)	0.532
Liver disease, *n* (%)	4 (16.0%)	4 (8.0%)	0.508
Neoplasia, *n* (%)	1 (4.0%)	2 (4.0%)	1.000
Splenomegaly, *n* (%)	2 (8.0%)	1 (2.0%)	0.532
HIV Characteristics			
AIDS, *n* (%)	15 (60.0%)	25 (50.0%)	0.567
HIV stage, *n* (%)			
A1–A3	9 (36.0%)	25 (50.0%)	0.367
B1–B3	8 (32.0%)	11 (22.0%)	0.511
C1–C3	8 (32.0%)	14 (28.0%)	0.929
Laboratory Values			
TPO (mg/mL), median (IQR)	114.7 (97.8–136.1)	142.7 (106.8–240.3)	0.047
Platelets (×10^9^/L), median (IQR)	109,000 (93,000–124,000)	139,000 (128,250–171,500)	0.002
Hemoglobin (g/dL), median (IQR)	12.7 (10.8–14.0)	13.2 (12.0–14.5)	0.136
White blood cells (×10^9^/L), median (IQR)	4670 (3040–5700)	5000 (3525–5775)	0.653
CD4 count (cells/µL), median (IQR)	185 (88–331)	253 (64.25–539.75)	0.360
Viral load (copies/mL), median (IQR)	71,600 (27,598–397,793.25)	192,000 (36,400–430,904)	0.601

Abbreviations: TPO: Thrombopoietin; *n*; number of participants in each group, IQR: interquartile range; HCV: hepatitis C virus; HBV: hepatitis B virus; HIV: human immunodeficiency virus; AIDS: acquired immunodeficiency syndrome; ITP: immune thrombocytopenia; CD4: cluster of differentiation 4.

**Table 4:** Alterations in the thrombocytopenia and hematological parameters before and after HAART initiation.

	Before HAART	After HAART	*p*-Value
Thrombocytopenia, *n* (%)	40 (75.47%)	18 (33.96%)	<0.001
Anti-TPO (+), *n* (%)	15 (28.30%)	9 (16.98%)	0.058
Platelets (×10^9^/L), median (IQR)	131,000.00 (103,000.00–148,000.00)	199,000.00 (137,000.00–254,000.00)	<0.001
Hemoglobin (g/dL), median (IQR)	13.40 (11.80–14.50)	13.80 (12.60–14.90)	<0.001
White blood cells (×10^9^/L), median (IQR)	4700.00 (3200.00–5510.00)	5700.00 (4600.00–6700.00)	<0.001
CD4 count (cells/µL), median (IQR)	142.00 (56.00–351.00)	430.00 (208.00–622.00)	<0.001
Viral load (copies/mL), median (IQR)	96,500.00 (31,367.00–323,391.00)	0.00 (0.00–0.00)	<0.001

Note: The longitudinal analysis was conducted on those patients who had anti-TPO measurements at both time points (*n* = 53). Abbreviations: *n*: number of participants in each group; IQR: interquartile range; TPO: thrombopoietin; CD4: cluster of differentiation 4.

**Table 5:** Median changes in platelet counts across each anti-TPO change category.

Category	N (Frequency)	Median Change in Platelet Count [IQR]
Change to Negativity	8	95,000 [43,750–199,500]
Change to Positivity	2	−25,000 [−36,000–(−14,000)]
Persistence of Negativity	36	58,000 [3250–106,750]
Persistence of Positivity	7	−9000 [47,500–(−53,000)]

**Table 6:** Changes in thrombocytopenia category proportions between time point 1 and time point 2.

Thrombocytopenia Category	Time Point 1 Proportion	Time Point 2 Proportion	*p*-Value
<50 k	0.500	1.000	0.361
50–99 k	0.375	0.800	0.135
100–150 k	0.321	0.273	0.767
≥150 k	0.077	0.028	0.443

## Data Availability

Data can be made available upon reasonable request to the corresponding author.
